# Impact of sleep problems on daytime function in school life: a cross-sectional study involving Japanese university students

**DOI:** 10.1186/s12889-020-08483-1

**Published:** 2020-03-20

**Authors:** Momoko Kayaba, Toshiko Matsushita, Minori Enomoto, Chieko Kanai, Noriko Katayama, Yuichi Inoue, Taeko Sasai-Sakuma

**Affiliations:** 1 Department of Somnology, Tokyo Medical University, 6-7-1 Nishishinjuku, Shinjuku-ku, Tokyo, 160-0023 Japan; 2 Japan Somnology Center, Institute of Neuropsychiatry, 5-10-10, Yoyogi, Shibuya-ku, Tokyo, 151-0053 Japan; 3 Department of Nursing, Graduate School of Medicine, Yokohama City University, Fukuura 3-9, Yokohama, Kanazawa-ku, Kanagawa 236-0004 Japan; 4 Department of Medical Technology, School of Health Sciences, Tokyo University of Technology, Nishikamata 5-23-22, Ota-ku, Tokyo, 144-0051 Japan; 5 Child Development and Education, Faculty of Humanities, Wayo Women’s University, Konodai 2-3-1, Ichikawa, Chiba, 272-0827 Japan; 6 Department of Nursing, Faculty of Medical Sciences, Shonan University of Medical Sciences, Kamishinano 16-48, Yokohama, Totsuka-ku, Kanagawa 244-0806 Japan; 7 Department of Clinical Laboratory Science, Faculty of Medical Technology, Teikyo University, Kaga 2-11-1, Itabashi-ku, Tokyo, 173-8605 Japan

**Keywords:** Insomnia, Behaviorally induced insufficient sleep syndrome, Delayed sleep-wake phase, Attendance, Tardiness, Daytime dysfunction

## Abstract

**Background:**

The aims of this study were 1) to clarify the prevalence of sleep problems (insomnia, insufficient sleep, and delayed sleep-wake phase) among Japanese university students; 2) to examine sociodemographic characteristics, lifestyle, and sleep-related symptoms in each sleep problem; and 3) to evaluate the association between the above-mentioned sleep problems and daytime dysfunction in school life.

**Methods:**

Self-report questionnaire surveys were conducted at eight universities in Japan, and we received 1034 valid answers (78% female). The questionnaire consisted of socio-demographic characteristics, information on lifestyle, sleep pattern, sleep-related symptoms, and daytime dysfunction in school life. Groups with insomnia, behaviorally induced insufficient sleep syndrome (BIISS), delayed sleep-wake phase (DSWP), and BIISS + DSWP were defined. To identify the association between sleep problems and daytime dysfunction in school life, the generalized linear mixed effect model was conducted.

**Results:**

Sleep duration on weekdays was 5.9 ± 1.2 h, and 38.2% of the students had a sleep duration < 6.0 h. About 16% of the students were categorized as evening-type individuals. More than half of the students (56.1%) had excessive daytime sleepiness. Insomnia was associated with tardiness (aOR: 0.8, 95%CI: 0.7–0.9) and falling asleep during class (aOR: 1.6: 95%CI: 1.4–2.0). BIISS was associated with tardiness (aOR: 1.5, 95%CI: 1.1–2.2) and interference with academic achievement (aOR: 1.9, 95%CI: 1.3–2.6). DSWP and BIISS + DSWP were associated with absence (aOR: 3.4, 95%CI: 2.2–5.1 / aOR: 4.2, 95%CI: 3.2–5.6), tardiness (aOR: 2.7, 95%CI: 1.8–4.1 / aOR: 2.2, 95%CI: 1.6–2.8), falling asleep during class (aOR: 2.6, 95%CI: 1.4–4.8 / aOR: 7.6, 95%CI: 3.3–17.2), and interference with academic achievement (aOR: 2.6, 95%CI: 1.7–3.9 / aOR: 2.1, 95%CI: 1.6–2.8).

**Conclusions:**

Students with DSWP and BIISS + DSWP were significantly associated with daytime dysfunction in school life, i.e. absence, tardiness, falling asleep during class and interference with academic achievement. Students displaying BIISS + DSWP were considered to have a relatively more serious condition compared with those with only insomnia, DSWP, or BIISS. It is therefore of utmost importance that university students aim to prevent DSWP and BIISS which were associated with daytime function in school life.

## Background

Sleep pattern and sleep problems vary depending on socio-demographic factors. Steptoe et al. reported that the average sleep duration for university students ranges from six to more than 8 hours in different countries, and no less than 21% of them have a short sleep duration (6%, < 6 h; 15%, 6–7 h) [1]. The study also revealed that the average sleep duration among university students in Asian countries including Japan (6.1 h), Taiwan (6.6 h), and Korea (6.8 h) were markedly shorter than those in other countries (over 7 h). In addition, the young population are likely to show the latest chronotype among all generations [2]; delayed endogenous circadian clock in the young population, school work, extracurricular activities, club activities, or part-time jobs may delay their bed time. Moreover, early awakening to adapt to their social schedule (e.g., for attending classes early in the morning) consequently brings about insufficient sleep especially in evening-type students. Numerous studies have reported that either insufficient sleep or later chronotype is associated with deterioration of physical health, mental health [3–5] and health-related quality of life [6, 7] as well as worse academic performance [8–12] in the young population.

It has been reported that as high as 33–59% of university students have poor sleep quality, which is associated with their worsened health status and academic performance [5, 13–19]. In these previous studies, sleep quality was defined as “poor” or “good” using the Pittsburg Sleep Quality Index (PSQI), a widely used scale evaluating the following seven components: subjective sleep quality, sleep latency, sleep duration, habitual sleep efficiency, sleep disturbances, use of sleep medication, and daytime dysfunction [20]. However, the PSQI score cannot determine what kind of sleep problems affect academic performance.

To improve students’ physical health, mental health, and academic performance, the American Academy of Pediatrics recommended a delayed school start time for adolescents [21]. Actually, several studies involving trials with delayed school start time recorded an increase in the sleep duration of students on weekdays, and consequently, improved mental health and daytime alertness in adolescents [22–24]. In university students, personal factors such as private activities rather than social schedule are likely to affect sleep duration because they do not have to keep a strict sleep-wake rhythm like junior / senior high school students who have to go to school on a regular schedule. Therefore, the characteristics of both university class schedules (e.g., school hours or class start time) and students’ lifestyle (e.g., commute time or living alone or with family) should be considered. Sleep problems, including insufficient sleep, insomnia, and sleep disorders, were categorized as “poor sleep” in previous studies. However, related factors and association with daytime dysfunction might differ among students with different sleep problems. Furthermore, few studies have evaluated daytime dysfunction in school life, such as absence, tardiness, and falling asleep during class, although test scores (e.g., grade point average) have been used to evaluate academic performance [8, 9, 12].

The aims of this study were 1) to clarify the prevalence of sleep problems (insomnia, insufficient sleep, and delayed sleep-wake phase) among Japanese university students; 2) to examine sociodemographic characteristics, lifestyle, and sleep-related symptoms in each sleep problem; and 3) to evaluate the association between the above-mentioned sleep problems and daytime dysfunction in school life.

## Method

### Participants and data collection

This cross-sectional study was conducted at eight universities in Japan between May 2018 and March 2019. Research collaborators from the fields of sleep science and psychiatric nursing were invited to participate in the study. Universities whose research collaborators both explained the survey and obtained ethical approval participated in this study. Research collaborators from the following university departments participated in the study: the faculty of nursing (4 universities), medical technology (2 universities), child development and education (1 women’s university), and science (1 university). Except for a university in the Tohoku region (*n* = 57), all the universities were located in the Kanto region (i.e. Tokyo, Kanagawa, Chiba). Self-reported questionnaires were distributed to undergraduate students during classes and were collected by means of a collection box or envelope so that answerers could not be identified, in accordance with the administrative regulation of the ethical approval committee in each university. We received 1069 questionnaires from eligible students (response rate, 78.3%) with 1034 valid answers, of which 225 of the respondents were male (21.8%) and 809 were female (78.2%). Twenty-two respondents were excluded from subsequent analyses according to exclusion criteria; no information on age, sex, or sleep pattern. Furthermore, students older than 22 years (*n* = 13) were excluded to limit age-dependent sleep variations within the “normal” age range of university students, i.e. 18–22 years.

### Measures

The questionnaire was written in Japanese and consisted of the following items.

#### Characteristics of the participants

For sociodemographic characteristics and lifestyle, students were required to answer questions on age, sex, grade, starting and closing time of class, whether they were living alone or not, commute time, club activities and/or part-time job, exercise habits per week (≥ 4 days /2–3 days/ ≤ 1 days/ none), alcohol consumption, smoking, self-rated health status, feeling stress, and use of sleep medication. Frequency of exercise habit was converted into a binary outcome (0: none, 1: ≥ 4 days /2–3 days/ ≤ 1 days) for analysis.

#### Sleep variables and definition of sleep problems

Data on sleep pattern included information on sleep duration, bedtime, awakening time on weekdays and on weekends, and extension of sleep duration on weekends. In addition to nocturnal sleep, we enquired about daytime nap (length and dreaming). The Japanese version of Epworth Sleepiness Scale (ESS) [25, 26] was used to assess the level of daytime sleepiness. The total sum score ranges from 0 to 24, and ≥ 11 is regarded as having excessive daytime sleepiness. Students indicated when their daytime sleepiness started as well. To assess chronotype, the Japanese version of Morningness-Eveningness Questionnaire (MEQ) [27, 28] was used. This questionnaire consists of 19 items, and a total score below 42 is defined as evening-type, 42–58 as neutral-type, and above 58 as morning-type. Sleep quality was assessed with the Japanese version of Athens Insomnia Scale (AIS) [29, 30], which indicates possible insomnia when one has a score above five out of the total score. In order to assess sleep-related symptoms, the self-report questionnaire included questions on whether the students had any insomnia symptoms (difficulty initiating sleep, difficulty maintaining sleep, and/or early morning awakening), difficulty awakening, REM sleep-related symptoms (hypnagogic hallucination and/or sleep paralysis), NREM parasomnias (night terrors, defective awakening, and/or sleep walking), and REM parasomnias (nightmare and/or acting out their dreams).

**Fig. 1:**
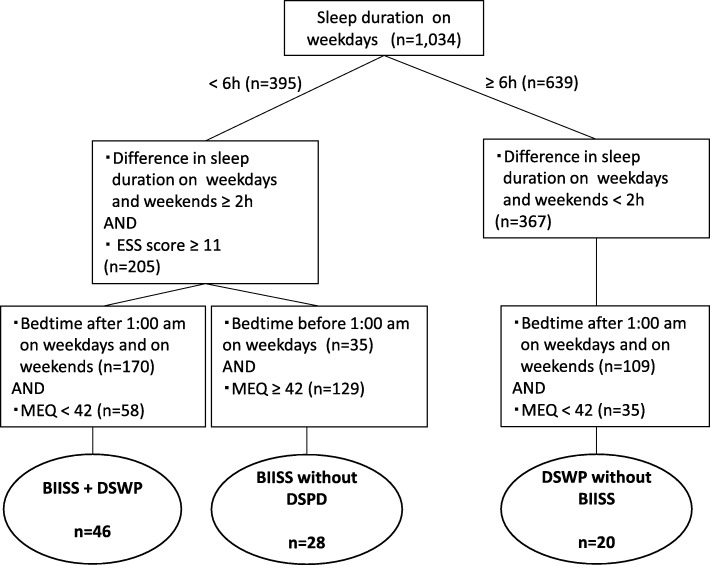
Definition of the BIISS, DSWP, and BIISS + DSWP groups. BIISS, behaviorally induced insufficient sleep syndrome; DSWP, delayed sleep-wake phase

In order to evaluate the association between sleep problems and daytime dysfunction in school life, we defined groups with insomnia, behaviorally induced insufficient sleep syndrome (BIISS), delayed sleep-wake phase (DSWP), and BIISS + DSWP as follows: Students who had an AIS score ≥ 6 and one or more of “Difficulty initiating sleep,” “Difficulty maintaining sleep,” and “Early morning awakening” were categorized as students with “Insomnia”. The groups with BIISS, DSWP, and BIISS + DSWP were categorized as shown in Fig. 1. Students whose sleep duration on weekdays was < 6 h, extension of sleep duration on weekends was ≥2 h, bedtime on weekdays was before 1 am, with MEQ score ≥ 42, and ESS score ≥ 11 were classified as having “BIISS”, the BIISS without DSWP group. Students whose sleep duration on weekdays was ≥6 h, extension of sleep duration on weekends was < 2 h, and bedtime on weekdays and weekends was after 1 am with MEQ score < 42 were classified as having “DSWP”, the DSWP without BIISS group. Students whose sleep duration on weekdays was < 6 h, extension of sleep duration on weekends was ≥2 h, bedtime on weekdays and weekends was after 1 am, with MEQ score < 42, and ESS score ≥ 11 were classified into the “BIISS + DSWP” group.

#### Daytime dysfunction in school life

The questionnaire included questions on frequency of absence, tardiness, and falling asleep during class (often/sometimes/none) and interference with academic achievement (yes/no). Frequency was converted into a binary outcome (0: none, 1: often/sometimes) for calculating odds ratio.

### Statistical analyses

Representative values were shown as mean ± standard deviation (SD). Mann-Whitney U tests and Chi-square tests followed by residual analyses were performed for continuous variables and categorical variables, respectively. To identify the association between sleep problems and daytime dysfunction in school life, the generalized linear mixed effect model was conducted. In the model, explanatory variables; sociodemographic characteristics, and lifestyle variables (sex, commute time, etc.) and sleep problems (presence/absence of insomnia, BIISS, DSWP, and BIISS + DSWP) were set as fixed effects, whereas university and grade were set as random effects. In the present study, insufficient sleep, delayed sleep phase, or combination of these two sleep problems were used as defined variables instead of original sleep parameters (e.g., sleep duration, MEQ score) in order to distinguish the impact of each sleep problem clearly. In the analysis of the generalized linear mixed effect model, the degree of association was represented as adjusted odds ratio (aOR) and 95% confidential interval (95%CI). These analyses involved multiple imputation by chained equations with a five-imputation dataset using logistic regression and a multinomial logit model for the categorical variables, as well as predictive mean matching for the numeric variables [31]. The significance level was defined as *p* < 0.05. Generalized linear mixed effect model analysis and multiple imputation by chained equations were performed using R statistical software version 3.5.1 (R Core Team, Vienna, Austria),“lme4” package [32] and “mice” package [33], and other analysis were performed by IBM SPSS Statistics 25 (IBM Corporation, Armonk, USA).

This manuscript was written in accordance with the STROBE statement for cross-sectional studies [34]. Ethical approval for this survey was granted by the Ethical Committee of the Tokyo Medical University and Institute of Neuropsychiatry.

## Results

### Characteristics of students

Characteristics of the study participants and their lifestyle data are shown in Table 1. Among the study participants there were more female (78.2%) than male students (21.8%). This study found that 21.1% of students lived alone. The results also found a mean commute time of 59.6 ± 33.9 min. Mean school hours were 6.9 ± 1.8 h (9:19–16:15). The percentage of the students who had club activities, part-time jobs, and exercise habits were 38.9, 66.3, and 48.3%, respectively. The average frequency per week was 1.7 ± 1.1 times for club activities and 2.6 ± 1.1 times for part-time jobs; 34.1% of the students with a part-time job worked after 11 pm. The percentage of the students who have alcohol consumption and smoking habits were 48 and 5.2%, respectively. The students who reported poor health and feeling stress accounted for 27.0 and 45.0% of the study population, respectively.

**Table 1:** Demographic characteristics and lifestyle data of the participants

	*n* = 1034
Age (y)	19.7 ± 1.2
Sex	
Male n (%)	225 (21.8)
Female n (%)	809 (78.2)
Grade	
Freshman n (%)	369 (35.7)
Sophomore n (%)	236 (22.8)
Junior n (%)	280 (27.1)
Senior n (%)	147 (14.2)
N/A n (%)	2 (0.2)
Living alone	
Yes n (%)	218 (21.1)
No n (%)	703 (68.0)
N/A n (%)	113 (10.9)
Commute time (min)	59.6 ± 33.9
School hours (h)	6.9 ± 1.8
Class start time (h:m)	9:19 ± 0:46
Class closing time (h:m)	16:15 ± 1:37
Club activities	
Yes n (%)	402 (38.9)
No n (%)	613 (59.3)
N/A n (%)	19 (1.8)
Part-time job	
Yes n (%)	686 (66.3)
No n (%)	333 (32.2)
N/A n (%)	15 (1.5)
Exercise habit	
Yes n (%)	499 (48.3)
No n (%)	519 (50.2)
N/A n (%)	16 (1.5)
Alcohol consumption	
Yes n (%)	437 (42.3)
No n (%)	584 (56.5)
N/A n (%)	13 (1.3)
Smoking	
Yes n (%)	54 (5.2)
No n (%)	967 (93.5)
N/A n (%)	13 (1.3)
Self-rated health status	
Good n (%)	740 (71.6)
Poor n (%)	279 (27.0)
N/A n (%)	15 (1.5)
Stress	
No n (%)	554 (53.6)
Yes n (%)	465 (45.0)
N/A n (%)	15 (1.5)

Values are expressed as mean ± standard deviation for continuous variables

*N/A* Unanswered

### Prevalence of sleep problems and sleep-wake pattern in Japanese university students

**Fig. 2:**
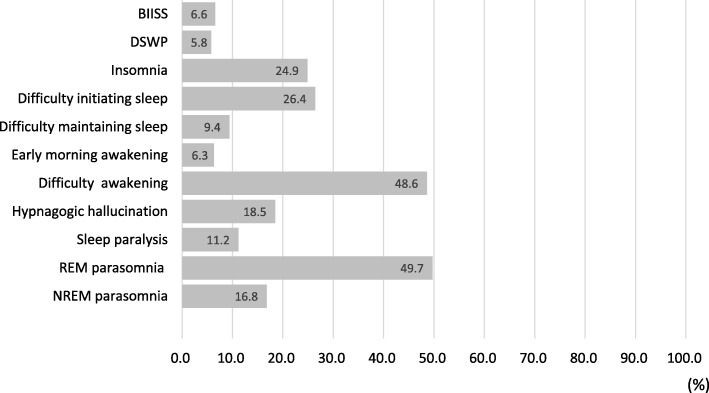
Prevalence of sleep problems and sleep-related symptoms. BIISS, behaviorally induced insufficient sleep syndrome: sleep duration on weekdays < 6 h, difference in sleep duration on weekdays and weekends ≥2 h, and Epworth Sleepiness Scale score ≥ 11. DSWP, delayed sleep-wake phase: bedtime before 1:00 am on weekdays and Morningness-Eveningness Questionnaire score < 42. Insomnia: Athens Insomnia Scale score ≥ 6 and one or more of “Difficulty initiating sleep,” “Difficulty maintaining sleep,” and “Early morning awakening”. REM parasomnia: nightmares and/or acting out dreams. NREM parasomnia: night terrors, defective awakening, and/or sleep walking

Habitual sleep schedules, chronotype, and presence of insomnia are summarized in Table 2. Sleep duration on weekdays was 5.9 ± 1.2 h, and 38.2% of the students had a sleep duration < 6.0 h. Extension of sleep duration on weekends for the students was 2.1 ± 1.7 h, and 55.8% of them had an extension of ≥2.0 h. According to the MEQ score, 16.2% of the students were categorized as evening-type individuals. The ESS score was 11.4 ± 4.6, and 56.1% of the students had excessive daytime sleepiness. Age at the self-reported onset of daytime sleepiness was 14.6 ± 3.1 years. The students who had AIS score ≥ 6 accounted for 52.6% of the total population, and 24.9% of the students met the criteria for insomnia. The prevalence of BIISS and DSWP were 6.6 and 5.8%, respectively (Fig. 2). In accordance with definitions (See *2.2.3*), 28, 20 and 40 students were categorized into the groups of BIISS, DSWP and BIISS+DSWP, respectively. Eight students (0.8%) reported sleeping medication taking. The prevalence of sleep-related symptoms is also shown in Fig. 2.

**Table 2:** Sleep-wake pattern in the groups with sleep problems

	Total (*n* = 1034)	Insomnia (*n* = 257)	BIISS (*n* = 28)	DSWP (*n* = 20)	BIISS + DSWP (*n* = 46)
Sleep duration on weekdays (h)	5.9 ± 1.2	5.7 ± 1.3	5.0 ± 0.5	6.8 ± 0.9	4.3 ± 1.0
Sleep duration on weekends (h)	8.0 ± 1.5	7.9 ± 1.6	8.5 ± 1.6	7.4 ± 1.1	8.6 ± 1.8
Bedtime on weekdays (h:m)	0:44 ± 1:07	0:57 ± 1:12	0:10 ± 0:22	1:34 ± 0:38	2:20 ± 1:01
Bedtime on weekends (h:m)	0:55 ± 1:24	1:06 ± 1:28	0:39 ± 2:11	1:51 ± 0:46	2:31 ± 1:20
Wake-up time on weekdays (h:m)	6:48 ± 1:03	6:44 ± 1:03	5:42 ± 0:42	8:31 ± 0:50	6:46 ± 0:54
Wake-up time on weekends (h:m)	9:02 ± 1:48	9:11 ± 1:57	9:21 ± 1:41	9:32 ± 0:53	11:20 ± 1:43
Extension of sleep duration on weekends (h)	2.1 ± 1.7	2.3 ± 2.0	3.5 ± 1.5	0.7 ± 0.6	4.4 ± 1.8
MEQ score	48.6 ± 7.5	47.9 ± 7.7	51.0 ± 6.2	37.1 ± 2.8	36.9 ± 4.3
Chronotype^a^					
Morning-type n (%)	95 (9.2)	22 (8.6)	4 (14.3)	0 (0)	0 (0)
Neutral-type n (%)	693 (67.0)	165 (64.2)	24 (85.7)	0 (0)	0 (0)
Evening-type n (%)	168 (16.2)	47 (18.3)	0 (0)	20 (100)	46 (100)
N/A n (%)	78 (7.5)	23 (8.9)	–	–	–
Athenes Insomnia Scale score	6.2 ± 3.7	9.7 ± 3.1	7.5 ± 3.7	6.3 ± 3.7	8.4 ± 3.6
Athenes Insomnia Scale score ≥ 6 n (%)	544 (52.6)	257 (100)	19 (67.9)	11 (55.0)	36 (78.3)
Insomnia^b^ n (%)	257 (24.9)	257 (100)	8 (28.6)	4 (20.0)	20 (43.5)
Epworth Sleepiness Scale score	11.4 ± 4.6	12.4 ± 4.4	14.6 ± 2.9	11.0 ± 4.0	14.7 ± 3.2
Epworth Sleepiness Scale score ≥ 11 n (%)	580 (56.1)	167 (65.0)	28 (100)	12 (60.0)	46 (100)
Sleep duration on weekdays < 6 h n (%)	395 (38.2)	119 (46.3)	28 (100)	0 (0)	46 (100)
Bed time after 1:00 am on weekdays n (%)	534 (51.6)	153 (59.5)	0 (0)	20 (100)	46 (100)

Values are expressed as mean ± standard deviation for continuous variables

*N/A* Unanswered

*MEQ* Morningness-Eveningness questionnarie

^a^Chronotype: total score for the Morningness-Eveningness questionnaire score below 42 is defined as evening-type, 42–58 as neutral-type, and above 58 as morning-type

^b^Insomnia: Atenes Insomnia Scale score ≥ 6 and one or more of “Difficulty in initiationg sleep”, “Difficulty in maintaining sleep”, and “Early morning awakening”

*BIISS* Behaviorally induced insufficient sleep syndrome, *DSWP* Delayed sleep-wake phase

### Sociodemographic characteristics, lifestyle and sleep-related symptoms in each sleep problem

Sociodemographic characteristics, lifestyle and sleep-related symptoms in each group of sleep problems were shown in Table 3. In the Insomnia group, age was higher, the students who had club activity were less prevalent, percentages of the students whose perceived health was poor and who had stress were higher than in the group without insomnia. Regarding nap habit, the percentages of the students who take a nap for over an hour and those who often dream during their nap were significantly higher than in the groups without insomnia. Regarding sleep-related symptoms, the percentages of the students with possible symptoms of not only insomnia symptoms (difficulty initiating sleep, difficulty maintaining sleep and early morning awakening) but also hypnagogic hallucination, sleep paralysis, REM parasomnia, and NREM parasomnia in the insomnia group were higher than in those without insomnia. In the BIISS group, all students were female and did not live alone. Commute time was longer, class start time was earlier, and the percentages of the students who had club activity and exercise habit were lower in the BIISS group than in the group without BIISS. In the DSWP group, the students who lived alone and had smoking habit were more prevalent, commute time and school hours were shorter, class start time was later than in the group without DSWP. Regarding sleep-related symptoms, difficulty awakening and NREM parasomnia were more frequently observed in the DSWP group than in the group without DSWP. In the BIISS + DSWP group, the percentage of the students who often dream during naps was significantly higher than in groups without BIISS + DSWP. Regarding sleep-related symptoms, possible positivity for difficulty initiating sleep, difficulty awakening, and hypnagogic hallucination was more frequently observed in the BIISS + DSWP group than in those without BIISS + DSWP.

**Table 3:** Sociodemographic and sleep characteristics and lifestyle in the groups with sleep problems

	Insomnia (*n* = 257)	Without Insomnia (*n* = 754)	BIISS (*n* = 28)	Without BIISS (*n* = 916)	DSWP (*n* = 20)	Without DSWP (*n* = 936)	BIISS + DSWP (*n* = 46)	Without BIISS + DSWP (*n* = 898)
Age (y)	19.8 ± 1.2	19.6 ± 1.2 ^a^	20.0 ± 1.2	19.7 ± 1.2	19.8 ± 1.0	19.7 ± 1.2	19.9 ± 1.0	19.7 ± 1.2
Sex								
Female (%)	79	78	100	79 ^a^	70	80	87	79
Grade								
Freshman (%)	31	37	25	36	25	36	20	36
Sophomore (%)	24	23	25	23	15	23	28	22
Junior (%)	30	27	32	27	50	27	37	27
Senior (%)	16	14	18	15	10	15	15	15
Living alone								
Yes (%)	21	21	0	22 ^a^	55	20 ^a^	17	21
Commute time (min)	59.6 ± 33.4	59.5 ± 34.4	85.4 ± 25.1	58.5 ± 33.5 ^a^	23.2 ± 20.0	60.0 ± 33.3 ^a^	66.2 ± 34.2	58.9 ± 33.5
School hours (h)	6.9 ± 1.9	7.0 ± 1.8	7.4 ± 1.4	7.0 ± 1.8	6.1 ± 1.6	7.0 ± 1.8 ^a^	6.8 ± 2.0	7.0 ± 1.8
Class start time (h:m)	9:18 ± 0:46	9:20 ± 0:46	8:59 ± 0:26	9:18 ± 0:45 ^a^	10:04 ± 1:11	9:17 ± 0:44 ^a^	9:22 ± 0:51	9:18 ± 1:36
Class closing time (h:m)	16:11 ± 1:39	16:17 ± 1:35	16:22 ± 1:22	16:15 ± 1:37	16:07 ± 1:57	16:15 ± 1:36	16:10 ± 1:39	16:16 ± 1:37
Club activities								
Yes (%)	32	41 ^a^	14	39 ^a^	45	38	22	39 ^a^
Part-time job								
Yes (%)	65	68	68	66	75	66	78	65
Exercise habit								
Yes (%)	46	49	25	49 ^a^	50	47	33	49 ^a^
Alcohol consumption								
Yes (%)	47	41	57	42	55	42	59	42 ^a^
Smoking								
Yes (%)	6	5	4	5	20	5 ^a^	4	5
Self-rated health status								
Poor (%)	41	22 ^a^	25	27	20	27	39	26
Stress								
Yes (%)	56	41 ^a^	46	45	45	45	65	44 ^a^
Duration of nap								
< 10 min (%)	27	31	31	30	20	30	15	31
10–29 min (%)	30	36	35	34	45	34	37	34
30–59 min (%)	22	19	19	19	10	20	24	19
≥ 1 h (%)	21	13 ^b^	15	15	25	15	24	15
Frequency of dreaming during nap								
Often (%)	18	12 ^b^	11	14	10	14	26	13 ^b^
Sometimes (%)	53	53	64	52	60	52	52	53
Not (%)	30	35	25	33	30	33	22	34
Sleep-related symptoms								
Difficulty initiating sleep (%)	76	10 ^a^	25	26	30	26	39	26 ^a^
Difficulty maintaining sleep (%)	24	5 ^a^	7	10	5	10	13	9
Early morning awakening (%)	15	4 ^a^	4	6	0	6	4	6
Difficulty awakening (%)	42	51 ^a^	43	49	80	49 ^a^	74	48 ^a^
Hypnagogic hallucination (%)	27	15 ^a^	32	18	15	19	35	18 ^a^
Sleep paralysis (%)	15	10 ^a^	11	12	15	11	20	11
REM parasomnia [7] (%)	57	47 ^a^	57	49	60	49	61	49
NREM parasomnia [8] (%)	24	15 ^a^	14	17	35	16 ^a^	24	16

^a^significantly difference between the students with the sleep problem and without the sleep problem

^b^significantly higher than other students by residual analysis (adjusted residual ≥1.96)

*BIISS* Behaviorally induced insufficient sleep syndrome, *DSWP* Delayed sleep-wake phase

*REM* Rapid eye movement, *NREM* Non-rapid eye movement

REM parasomnia: nightmare and / or acting out their dreams

NREM parasomnia: night terrors, defective awakening, and / or sleep walking

### Factors associated with daytime dysfunction in school life

The percentage of the students who reported “Often”, “Sometimes”, and “None” were 29 (3%), 175 (17%), and 822 (80%) for absence; 40 (4%), 256 (25%), and 727 (70%) for tardiness; and 175 (17%), 666 (64%), and 179 (17%) for falling asleep during class, respectively. Further, 241 (23.3%) students reported interference with academic achievement. Factors associated with daytime dysfunction in school life are shown in Table 4. Insomnia was associated with tardiness (aOR: 0.8, 95%CI: 0.7–0.9) and falling asleep during class (aOR: 1.6: 95%CI: 1.4–2.0). BIISS was associated with tardiness (aOR: 1.5, 95%CI: 1.1–2.2) and interference with academic achievement (aOR: 1.9, 95%CI: 1.3–2.6). DSWP and BIISS + DSWP were associated with absence (aOR: 3.4, 95%CI: 2.2–5.1 / aOR: 4.2, 95%CI: 3.2–5.6), tardiness (aOR: 2.7, 95%CI: 1.8–4.1 / aOR: 2.2, 95%CI: 1.6–2.8), falling asleep during class (aOR: 2.6, 95%CI: 1.4–4.8 / aOR: 7.6, 95%CI: 3.3–17.2), and interference with academic achievement (aOR: 2.6, 95%CI: 1.7–3.9 / aOR: 2.1, 95%CI: 1.6–2.8).

**Table 4:** Generalized linear mixed effect model^b^ for associated factors with daytime dysfunction in school life

	Absence		Tardiness		Falling asleep during class		Interference with academic achievement	
	Often, sometimes (*n* = 204) / None(*n* = 822)		Often, sometimes (*n* = 296) / None(*n* = 727)		Often, sometimes (*n* = 841) / None(*n* = 179)		Yes (*n* = 241) / No(*n* = 751)	
Explanatory variables	Crude OR (95%CI)	aOR^a^ (95%CI)	Crude OR (95%CI)	aOR^a^ (95%CI)	Crude OR (95%CI)	aOR^a^ (95%CI)	Crude OR (95%CI)	aOR^a^ (95%CI)
Sex (Female)	0.5 (0.4–0.6)^**^	0.6 (0.5–0.7)^**^	0.6 (0.5–0.7)^**^	0.9 (0.8–1.1)	2.1 (1.8–2.4)^**^	2.1 (1.7–2.6)^**^	1.1 (0.9–1.3)	1.3 (1.0–1.6)^*^
Class start time (h)	1.1 (1.0–1.2)	1.1 (1.0–1.2)^*^	1.2 (1.1–1.3)^**^	1.1 (0.9–1.2)	1.0 (0.9–1.1)	1.0 (0.9–1.1)	0.9 (0.8–0.9)^*^	0.8 (0.8–0.9)^*^
Class closing time (h)	1.0 (0.9–1.1)	1.0 (0.9–1.0)	0.9 (0.9–0.9)*	0.9 (0.8–0.9)^**^	1.1 (1.0–1.1)^*^	1.0 (0.9–1.1)	1.1 (1.1–1.2)^**^	1.0 (0.9–1.1)
Living alone	1.9 (1.7–2.2)^*^	1.5 (1.2–1.8)^**^	2.2 (1.9–2.5)^**^	1.5 (1.3–1.8)^**^	0.8 (0.7–0.9)^*^	1.3 (1.0–1.6)^*^	1.2 (1.1–1.4)^*^	1.4 (1.1–1.6)^*^
Commute time (h)	0.7 (0.6–0.8)^**^	0.9 (0.8–1.1)	0.6 (0.6–0.7)^**^	0.8 (0.7–0.9)^*^	1.5 (1.3–1.7)^**^	1.7 (1.4–2.0)^**^	1.1 (0.9–1.2)	1.3 (1.1–1.5)^*^
Club activities	0.7 (0.6–0.8)^**^	0.4 (0.4–0.5)^**^	1.2 (1.1–1.4)^*^	0.9 (0.7–1.1)	1.0 (0.9–1.2)	1.2 (1.0–1.5)	1.0 (0.8–1.1)	0.9 (0.8–1.1)
Part-time job	1.7 (1.4–1.9)^**^	1.9 (1.6–2.2)^**^	1.8 (1.6–2.1)^**^	2.1 (1.8–2.5)^**^	2.4 (2.1–2.8)^**^	2.2 (1.8–2.5)^**^	1.4 (1.3–1.7)^**^	1.3 (1.1–1.5)^*^
Exercise habit	1.4 (1.2–1.6)^**^	1.5 (1.3–1.8)^**^	1.4 (1.2–1.6)^**^	1.3 (1.1–1.5)^*^	0.8 (0.7–0.9)^**^	0.8 (0.7–0.9)^*^	1.0 (0.9–1.1)	1.3 (1.1–1.5)^*^
Alcohol consumption	1.5 (1.3–1.7)^**^	1.4 (1.1–1.7)^*^	1.4 (1.2–1.6)^**^	1.2 (1.0–1.4)^*^	1.4 (1.2–1.6)^**^	1.6 (1.3–2.0)^**^	1.5 (1.3–1.7)^**^	1.0 (0.8–1.1)
Smoking habit	2.3 (1.8–3.0)^**^	1.7 (1.2–2.3)^*^	1.9 (1.5–2.4)^**^	1.5 (1.1–1.9)^*^	0.7 (0.5–0.9)^*^	0.5 (0.4–0.8)^**^	1.5 (1.1–1.9)^*^	1.2 (0.9–1.6)
Poor health	1.2 (1.0–1.4)^*^	1.2 (0.9–1.4)	1.4 (1.2–1.6)^**^	1.6 (1.3–1.8)^**^	1.2 (1.0–1.4)^*^	1.1 (0.9–1.3)	2.4 (2.1–2.8)^**^	1.9 (1.6–2.2)^**^
Stress	1.2 (1.0–1.3)^*^	1.1 (0.9–1.3)	1.3 (1.2–1.5)^**^	1.3 (1.1–1.5)^*^	1.2 (1.0–1.4)^*^	1.1 (0.9–1.3)	2.5 (2.2–2.8)^**^	1.9 (1.6–2.2)^**^
Sleep medication	3.6 (1.9–6.6)^**^	5.0 (2.6–9.7)^**^	0.7 (0.3–1.5)	0.8 (0.4–1.8)	1.3 (0.6–3.5)	1.4 (0.6–3.4)	1.9 (0.9–3.4)	2.0 (1.0–4.0)^*^
Insomnia	1.2 (1.0–1.4)^*^	1.1 (0.9–1.3)	0.9 (0.8–1.0)	0.8 (0.7–0.9)^*^	1.6 (1.3–1.9)^**^	1.6 (1.4–2.0)^**^	1.4 (1.2–1.6)^**^	1.1 (0.9–1.3)
BIISS	0.9 (0.6–1.3)	1.1 (0.7–1.7)	1.1 (0.8–1.6)	1.5 (1.1–2.2)^*^	1.1 (0.7–1.7)	0.7 (0.5–1.1)	2.0 (1.4–2.7)^*^	1.9 (1.3–2.6)^**^
DSWP	3.5 (2.4–5.1)^*^	3.4 (2.2–5.1)^**^	3.5 (2.4–5.0)^**^	2.7 (1.8–4.1)^**^	1.8 (1.0–3.4)^*^	2.6 (1.4–4.8)^*^	1.9 (1.3–2.8)^*^	2.6 (1.7–3.9)^**^
BIISS+DSWP	3.9 (3.0–5.0)^*^	4.2 (3.2–5.6)^**^	2.1 (1.6–2.7)^**^	2.2 (1.6–2.8)^**^	9.6 (4.6–24.3)^*^	7.6 (3.3–17.2)^**^	2.6 (2.0–3.3)^**^	2.1 (1.6–2.8)^**^
**Random Effects**	Variance		Variance		Variance		Variance	
University n = 8	0.3		0.2		0.1		0.1	
Grade n = 4	0.1		0.0		0.1		0.2	
Observation	5170 ^(2)^							
Conditional R^2^	0.2		0.2		0.2		0.2	

*BIISS* Behaviorally induced insufficient sleep syndrome, *DSWP* Delayed sleep-wake phase

*OR* Odds ratio, *CI* Confidencial interval, *aOR* Adjusted odds ratio

* *p* < 0.05, ** *p* < 0.001

^a^aOR indicated OR adjusted for all other explanatory variables

^b^Multiple imputation by chained equations with a five-imputation dataset was performed for missing data

## Discussion

### Insomnia, insufficient sleep and delayed sleep-wake phase in university students

The present study revealed the current statuses of university students with regard to insomnia, insufficient sleep, and delayed sleep-wake phase. In a previous systematic review on insomnia in university students, the prevalence of insomnia ranged from 9.4 to 38.2% [35–37]. The prevalence of insomnia among the university students included in the present study fell within this range. In the present study, 24.9, 2.7, 1.9, and 4.4% of students were categorized as having insomnia, BIISS (without DSWP), DSWP (without BIISS), and BIISS + DSWP, respectively. In contrast to the results of previous studies in which short sleep was judged only by the cut-off value of sleep duration (e.g., < 6 h) [1, 4, 8], we defined BIISS by including the following criteria; presence of daytime sleepiness and oversleeping on weekends. Likewise, DSWP was defined by assessing bedtime and MEQ score. Sleep duration and delayed sleep phase are correlated [38]; however, a number of previous studies that evaluated the impact of late chronotypes did not consider sleep duration [3, 39, 40]. Therefore, we defined BIISS, DSWP, and BIISS + DSWP more strictly by following the aforementioned criteria in order to investigate the actual impact of insufficient sleep and delayed sleep-wake phase. As a result, the prevalence of BIISS was less than 10% in the present study; however, about half of the total participants were suspected to have insufficient sleep because 38% of them reported that their sleep duration was < 6 h on weekdays and half of them extended their sleep duration by 2 h on weekends and/or showed ESS score ≥ 11. Similarly, the actual prevalence of delayed sleep-wake phase could also be higher than that reported in the present study (6%) because 16% of the students were categorized as evening-type individuals. The sleep duration of students in our study was short; this is in line with the results of previous studies, which reported that the sleep duration of Japanese university students was shorter than those of university students in other countries [1]. In the present study, the prevalence of evening-type individuals (16%) was lower than that of previous studies, which reported results that ranged from 20 to 35% [41–43].

### Characteristics of each sleep problems

The present study provided the characteristics of the aforementioned sleep problems considering not only participants’ sociodemographic information or lifestyle but also their sleep-related symptoms. Although their sleep-related symptoms were not confirmed by face to face interview or validated screening scale, this is the first study to investigate these symptoms and their associations with sleep problems.

The prevalence of REM related symptoms such as hypnagogic hallucination and sleep paralysis were 19 and 11%, respectively. In the general population, the prevalence of hypnagogic hallucination was 31% in the 15–44 age group and the symptom was associated with mental health disorders including anxiety disorders, depression disorders, and short sleep duration [44]. In contrast, the prevalence of sleep paralysis was 2% in the 15–24 age group [45], and 13% in university students [46] and the symptom was also associated with mental/physical disease, non-restorative sleep, nocturnal leg cramps, and nightmares [45, 47]. In the present study, the prevalence of NREM/REM parasomnia was 17 and 50%, respectively. NREM/REM parasomnia are likely to be associated with insufficient sleep and a delayed sleep phase. In the case of REM parasomnia, nightmares are induced by stressful or traumatic events and are linked to insomnia [48]. Taking these results into account, these sleep-related symptoms may cause sleep problems or mental/physical dysfunction in the young adult population.

Hypnagogic hallucination (27%), sleep paralysis (15%), and REM/NREM parasomnia (57%/24%) other than insomnia symptoms (i.e., difficulty initiating sleep, difficulty maintaining sleep, early morning awakening) were more prevalent in the insomnia group than in the students without insomnia. This is consistent with the results of previous studies, which reported that hypnagogic hallucination was most frequent in individuals who had difficulty initiating sleep [49]. In the insomnia group, the percentage of the students who took a nap for over an hour was significantly higher than that in the students without insomnia. This may be as a result of unsatisfactory nocturnal sleep due to insomnia. Contrarily, taking a long nap during the day may disturb the sleep-wake rhythm and evoke hypnagogic hallucination. In a previous study, forced two-hour nap in the early evening shortened REM latency and worsened sleep efficiency following nocturnal sleep in young adults [50]. Generally, a short nap has a positive effect on daytime performance and reduces fatigue; therefore, appropriate timing and length of naps should be considered [51].

In the BIISS group, the prevalence of sleep-related symptoms did not differ from that recorded in the students without BIISS, despite the longer commute and earlier class start times. Interestingly, all students with BIISS were female who did not live alone. Thus, for these university students, it was suggested that insufficient sleep might be affected by external environmental factors.

Difficulty awakening (80%) and NREM parasomnia (35%) were more prevalent in the DSWP group than in the students without DSWP. The high prevalence of difficulty awakening in this group was reasonable because the students with DSWP needed to wake up earlier than their internal clock time. In contrast to the BIISS group, commute time was shorter and school start time later in the DSWP group than in the group without DSWP. Students with short commutes and late school start times were generally on time for school even though they wake up relatively later due to their age-dependent eveningness. Furthermore, it is possible that students with DSWP take late start classes as a coping strategy; that is, these two school lifestyles can be either cause DSWP or be an effect of it.

In the BIISS + DSWP group, difficulty initiating sleep (39%), difficulty awakening (74%), and hypnagogic hallucination (35%) were more prevalent than in the students without BIISS + DSWP. In contrast to the students with only DSWP who could have a sufficient length of nocturnal sleep, the students with BIISS + DSWP would be forced to get up early. Moreover, their bedtime was quite late (2:20 ± 1:01) and one fourth of them had difficulty initiating sleep. Given this, the BIISS + DSWP group included the students who have an evening-type chronotype due to their delayed internal clock but could wake up early in order to adjust to their social schedule. Therefore, students with BIISS + DSWP, most of whom are thought to be unaware of their delayed intrinsic circadian rhythm, may have profoundly worsened health in the long run.

### The association between sleep problems and daytime dysfunction in school life

Delayed sleep-wake phase (DSWP and BIISS + DSWP) were associated with all instances of daytime school life dysfunction, i.e., absence, tardiness, falling asleep during class, and interference with academic achievement, whereas BIISS was associated with only tardiness and interference with academic achievement. Compared to DSWP, BIISS + DSWP showed higher aOR for absence and falling asleep during class. In students with BIISS + DSWP, most are thought to suffer from serious insufficient sleep as a result of being forced to get up early, despite their delayed internal clock. Thus, BIISS + DSWP is considered a relatively more serious condition compared to DSWP. Absence and tardiness may be caused by difficulty awakening in the morning. Absence is considered to be a more serious condition than tardiness, which was negatively associated with being female (aOR: 0.6). This finding is consistent with the previous report stating that men are more likely to show biological eveningness [2]. In this study, a quarter of the students internal clocks were considered delayed due to their difficulty in initiating sleep, many of whom may be unaware of their delay. Therefore, in order to prevent the acceleration of this phase delay, it is important that university students learn about age-dependent sleep problems i.e. delayed sleep-wake phase and resulting insufficient sleep, and to avoid staying up late and/or extending their sleep duration on weekends. In this present study, results showed that over 80% of the students had experienced falling asleep during class. Insomnia (aOR: 1.6), DSWP (aOR: 2.6) and BIISS + DSWP (aOR: 7.6) were all associated with falling asleep during class. The BIISS + DSWP group reported severe insufficient sleep i.e. 4.3 h on weekdays, and only 4.4 h on weekends, with a delayed sleep-wake phase i.e. 2:20 am weekday bedtime. Key aspects to improve university student daytime function are thus maintaining a regular sleep-wake cycle and extending sleep duration. Students who cannot improve daytime sleepiness and difficulty awakening even after these attempts should be examined for sleep disorders, such as circadian rhythm disorders, narcolepsy, sleep apnea syndrome, or restless legs syndrome. Association was confirmed between interference with academic achievement and BIISS (aOR: 1.9), DSWP (aOR: 2.6), and BIISS + DSWP (aOR: 2.1). This result coincided with the findings of previous studies that reported a similar association between sleep problems and worsened academic performance e.g. grade point or ranking [8, 9, 12].

Some previous reports emphasized that delay of class start time improved academic outcomes in adolescents because of the consequent adjustment in their internal clocks [52, 53]. In contrast, other studies reported that late class start times possibly lead to alcohol consumption and may have a negative influence on academic performance in university students [54]. Unlike adolescents (junior/senior high school students), university students can engage in activities such as alcohol consumption and working a part time job in the night, freely and without supervision. In this study, students who displayed alcohol consumption and smoking habits were more prevalent in the BIISS + DSWP and DSWP groups respectively. Although alcohol consumption and smoking are prohibited under the age of 20 by law in Japan, these students were not excluded from this study. Thus, the lower percentage of students in this study whom consume alcohol and smoke compared to other countries may thus be due to this law. Therefore, although we cannot conclude the association and causal relationship from this cross-sectional study without adjusting for confounding factors, a delayed sleep-wake phase might be influenced not only by students’ internal clock, but also by their lifestyle habits. Further studies should clarify whether BIISS + DSWP is associated with alcohol consumption itself or with the drinking habits are necessary. Furthermore, having a part-time job was associated with absence, tardiness, falling asleep during class, and interference with academic achievement, independent of sleep problems. Further studies should investigate the reasons for which students engage in part-time work and the detailed schedules of students with part-time jobs.

In this study, sleep medication was associated with absence (aOR: 5.0) and interference with academic achievement (aOR: 2.0). It was unclear why participants took sleep medication. However, it can be assumed that students with worse physical/mental health status might be prescribed sleep medication at the hospital, as indicated by the associations between poor health and tardiness or interference with academic achievement, and between stress and interference with academic achievement. However, this cross-sectional study could not draw causal inferences due to the small number (*n* = 8) of students on sleep medication. A longitudinal study is thus needed in order to better understand the association between daytime dysfunction in school life and physical/mental health including sleep medication use.

### Study limitations

This study had some limitations. First, differential diagnosis of sleep problems / sleep-related symptoms could not be done in this questionnaire-based study. Second, universities were not randomly selected, because survey data was collected from the universities where the research collaborators were able to directly contact the ethics committee. Therefore, our study sample may not be representative of Japanese university students and sampling bias might exist especially since the percentage of female students was high; this was because women’s university and faculties (e.g., nursing) participated in this study. Eveningness is more severe in males [2]; therefore, the impact of sleep problem on school life may be stronger in male students. Third, the causal relationship between sleep problems and daytime function in school life could not be ascertained in this cross-sectional study. Further studies on prospective sleep evaluation using objective sleep parameters are needed. In addition, although the questionnaire inquired about sleep-related symptoms and daytime function in school life, due to the lack of a time-frame measurement, misleading associations could have been found. Fourth, sleep-related symptoms could not be diagnosed using the validated questionnaires. Finally, the potential impact of covariates that were not included in our analyses should be considered, because various social or lifestyle factors may influence both sleep problems and daytime dysfunction in school life.

## Conclusions

Students with DSWP and BIISS + DSWP were significantly associated with daytime dysfunction in school life, i.e. absence, tardiness, falling asleep during class and interference with academic achievement. Students displaying BIISS + DSWP were considered to have a relatively more serious condition compared with those with only insomnia, DSWP, or BIISS. It is therefore of utmost importance that university students aim to prevent DSWP and BIISS which were associated with daytime function in school life.

## Data Availability

The datasets used and/or analyzed during the current study are available from the corresponding author on reasonable request.

## Abbreviations

AIS: Athens Insomnia Scale
BIISS: Behaviorally induced insufficient sleep syndrome
DSWP: Delayed sleep-wake phase
ESS: Epworth Sleepiness Scale
MEQ: Morningness-Eveningness Questionnaire
NREM: Non-rapid-eye movement
REM: Rapid-eye movement
PSQI: Pittsburg Sleep Quality Index
